# The potential of spot urine as a biomarker for zinc assessment in Malawian children and adults

**DOI:** 10.3389/fnut.2022.890209

**Published:** 2022-07-22

**Authors:** Blessings H. Likoswe, R. Murray Lark, John Phuka, Kenneth Maleta, Edward Joy, Nicola M. Lowe

**Affiliations:** ^1^Department of Nutrition and Dietetics, School of Global and Public Health, Kamuzu University of Health Sciences, Blantyre, Malawi; ^2^School of Biosciences, University of Nottingham, Loughborough, United Kingdom; ^3^Faculty of Epidemiology and Population Health, London School of Hygiene and Tropical Medicine, London, United Kingdom; ^4^UCLan Research Centre for Global Development, University of Central Lancashire, Preston, United Kingdom

**Keywords:** biomarker, micronutrient, urine zinc, serum zinc, spot urine, inflammation, hydration

## Abstract

Population-level assessment of zinc deficiency remains a challenge due to the lack of suitable biomarkers. Spot urinary zinc concentration (UZC) has the potential to provide information on population zinc status in large-scale surveys, but there is no established cut-off point indicating deficiency. A strong correlation between this biomarker and an established biomarker such as serum zinc concentration (SZC) in paired samples (i.e., from the same individual), could identify the thresholds indicating zinc deficiency. This study, therefore, aimed to regress spot UZC from school-aged children and women from the Malawi micronutrient survey with paired SZC data using a linear mixed-effects model. The nested variance components indicated no linear relationship between the UZC and SZC data, irrespective of adjustments for inflammation and hydration. Thresholds of urinary zinc excretion that have been suggested by expert panels were applied to the spot UZC data, as a *post-hoc* analysis. The zinc deficiency prevalence estimates derived from these suggested thresholds were not similar to the estimates from the SZC data, and further research is required to understand whether spot UZC can still provide useful information in population zinc assessment.

## Introduction

Zinc is an essential micronutrient, required in the diet in relatively small amounts, yet it is involved in many metabolic and physiological processes. Biochemical data on zinc deficiency are sparse, however, the limited data on population-level analyses of plasma or serum zinc (SZC) levels have shown that zinc deficiency is of global public health concern (1). Zinc deficiency may lead to poor immunity and greater susceptibility to infections. It can also cause poor growth, poor wound healing, birth defects, and death in infants (2). Zinc deficiency underlies a substantial disease burden predominantly among children under 5 years of age. It affects one in every two children from low- and middle-income countries and is associated with reduced linear growth as well as increased incidences of infectious diseases (3).

Plasma or SZC is used as a biomarker to assess population-level zinc status but it has limitations (2, 4) including confounding factors such as acute inflammation which cause artificial inflation of the prevalence estimates (5–8). Adjustments can be made to account for the effects of inflammation, however, the existing methods are not consistent with each other (9, 10). There are currently three established methods that are used to correct for inflammation but the prevalence estimates that are produced by these methods differ. Other biomarkers such as nails, hair, and sweat, have been proposed but are not routinely used due to multiple limitations such as biological factors and environmental contamination (2, 11). Single-void (spot/casual), urine is an established biomarker that is used for population-level assessment of iodine (12), and it has shown value in the population-level assessment of selenium (13). It is thus a good candidate for assessing population zinc status on a large scale but there are no established cut-off values for spot urinary zinc concentration (UZC) which limits its usefulness. Prior studies have used a factorial approach to calculate the physiological requirements of zinc in healthy participants, which involves identifying typical values for urinary zinc losses in healthy individuals by age range (14, 15). These values may be useful in establishing cut-off points; however, they were based on 24-h urinary samples and may not be appropriate for spot urine samples.

A strong correlation between paired data of an established biomarker with known cut-offs indicating deficiency and a surrogate with no known cut-offs can be used to identify thresholds for deficiency in the latter (16, 17). This method is used in blood serum biomarkers for vitamin A deficiency, but random, or “spot” urine samples have also been useful surrogates for other nutrients. For instance, spot urine selenium has been identified as a strong candidate for determining population-level selenium status in specific demographic groups because of its strong correlation with paired-sample serum selenium concentration (13). We, therefore, hypothesized that a strong correlation between UZC and SZC could also be used to determine cut-off points for UZC that are equivalent to those in SZC. The objectives of this study were (1) To assess the strength of the relationship between UZC data against SZC data in the Malawi Micronutrient Survey population using linear mixed-effects modeling; (2) If a strong correlation between SZC and UZC data is found to identify the optimal cut-off point in UZC for estimating the risk of zinc deficiency.

## Materials and methods

We used secondary data from the Malawi National Micronutrient Survey (MNS) which was conducted from 2015 to 2016. The study design has been previously reported in detail (18) and is presented here in brief. The survey used a two-stage cluster design which aimed to provide results weighted at national, regional (north, central and south), district (*n* = 28), and residency (rural/urban) levels. This was done using household-level sampling weights, which were derived from the Malawi Demographic Health Survey. The sampling method resulted in a total of 105 clusters (35 in each of the regions) from which households were selected for inclusion in the MNS study (20 per urban cluster and 22 per rural cluster). In each household, all primary school children (PSC) were included, and from nine random households of the 20/22 households, all women of reproductive age (WRA) were included. Of the nine random households, six were randomly selected for inclusion of all school-age children (SAC), and from these six households, four were included for adult men.

The sample size taken for this current study was determined by the availability of the urine data, which was determined by the sample availability. The spot urine samples were only collected from WRA and SAC for the assessment of iodine concentrations and other conditions. The urine sample collection method is described in detail in an earlier publication (13), while sample collection and analysis of the SZC are detailed in the MNS report (18). Serum samples for zinc assessment were collected in trace element-free vacutainers. The samples were maintained in portable freezers in the field and transported to the nearest district laboratory for temporary storage (−20°C). The samples were then transferred to a central laboratory and stored at −70°C until shipment to CHORI lab in Oakland, for analysis. In the lab, serum zinc was diluted to a final concentration of 5% HNO_3_ and centrifuged at 3,000 × g for 10 min before analysis using Atomic Emission Spectrometry (AES) (19, 20).

Subsequently, zinc concentrations were measured in stored urine samples, as reported previously (21). In brief, the urine samples were frozen within an hour of collection and kept at −20°C for a maximum of 8 h, then they were transferred to a central laboratory in Malawi which stored them at −80°C (13). The samples were then transferred frozen and on dry ice, to the UK where they were also kept at −80°C until the time of sample analysis. Zinc concentration was determined in a multi-element analysis (with a Helium collision mode, to reduce interferences) using the Inductively Coupled Plasma Mass Spectrometry (ICP-MS) assay (21). The limit of detection was 19 μg/L based on the standard deviation of 137 analytical blanks. Certified reference materials of human urine and seronorm trace elements of urine were used as quality controls for the analysis. In total, zinc concentration was measured in 1,406 urine samples (*n* = 741 and *n* = 665 participants for WRA and SAC, respectively). This study included only observations that had serum zinc data, inflammation adjustment data, and urinary data.

The UZC data were adjusted for effects of hydration (see Section Adjustment of urine data for hydration) before running the linear-mixed effects models. The SZC data used in the model was adjusted for inflammation (Section Adjustment of serum zinc data for inflammation) in a prior study (9).

### Adjustment of urine data for hydration

Creatinine adjustment was conducted using the equation below


$$\begin{array}{l} UZC_{cr}=\;\frac{UZC_{unadj\;}}{Cr_{meas}} \end{array}$$

Where UZC_cr_ denotes adjusted UZC and was reported as UZC_cr_ μg/L, UZC_unadj_ denotes unadjusted UZC, and CR_meas_ denotes, the measured creatinine concentration of the individual.

Osmolarity adjustment was achieved by the equation


$$\begin{array}{l} UZC_{osm}=\;UZC_{unadj}\;*\;\frac{OSM_{mean}}{OSM_{meas}} \end{array}$$

Where UZC_osm_ denotes osmolarity-adjusted UZC and is reported as UZC_osm_ μg/L, UZC_unadj_ denotes the unadjusted UZC, OSM_mean_ denotes the mean osmolarity from all the data and OSM_meas_ denotes the individual osmolarity measurement.

These adjustments were all made separately for the WRA and SAC data and resulted in four different UZC data points for each individual (unadjusted, Sg adjusted, creatinine adjusted, and osmolarity adjusted). A urine sample quality check was made to identify samples that were too dilute for inclusion in the analysis. This was conducted using a combined cut-off mark of creatinine level ≤ 0.05 g/L and an Sg of ≤ 1.001 (22).

### Adjustment of serum zinc data for inflammation

Biomarkers of inflammation C-reactive protein (CRP) and alpha-1 acid glycoprotein were analyzed in serum samples that were aliquot from the blood collected in all participants. This analysis was conducted using the sandwich Enzymatic Linked Immunosorbent Assay (ELISA) at the VitMin lab in Germany (18). Three forms of the serum zinc data were used for the analyses: (1) Raw uncorrected data (SZC_unadj_ μg/dl), (2) corrected for inflammation using internal correction factors (ICF, SZC_icf_ μg/dl), and (3) corrected for inflammation using Biomarkers Reflecting Inflammation and Nutritional Determinants of Anemia (BRINDA, SZC_br_ μg/dl) recommended methods (5, 6, 23).

### The linear mixed-effects model

The relationship between UZC and SZC was examined by fitting a linear model. Because of the clustered structure of the observations, a linear mixed-effects model (LMM) was used with random effects to account for nested variation at three levels: between cluster, within-cluster (between households), and within households (between individuals). Log-transformed data were used for both SZC and UZC to make the assumption of normality of the random effects plausible.

The basic LMM takes the form


y=Xβ+η+κ+ε

where the vector contains observation of the dependent variable, the design matrix **X** contains covariates and the vector **β** contains the corresponding fixed effects coefficients, and the terms **η**, **κ**, and **ε** are, respectively, the between-cluster, between household (within the cluster), and between individual (within the household) random effects. Each of the random effects has a mean of zero and a variance that must be estimated.

In the so-called null model, the only fixed effect is a constant mean, so the design matrix **X** contains a single column, with values all 1, and the vector **β** contains the estimated mean value. We used the lme function from the nlme package in R (24) to estimate the variances of the random effects by residual maximum likelihood. In an alternative model, UZC is considered as a potential predictor for SZC, and so it is a fixed effect in the model, along with a constant intercept. In this case, the design matrix **X** comprises a column, with values all 1, and a second column that contains the values of UZC. The vector **β** contains a constant or intercept and the regression coefficient for UZC in the estimated model. The variance components from the predictor model were then compared with those from the null model to determine the magnitude of reduction that was introduced by the addition of UZC data to the model. The predictor model also gave adjusted *R*^2^, at the nested levels which are similar to the standard *R*^2^ but not guaranteed to be bound to the interval (0, 1). A higher adjusted *R*^2^ at the cluster level than the individual level would imply that UZC may have greater value as a population-level, rather than individual-level, biomarker, and vice versa. For the models which gave such outputs, cluster means of the two datasets were plotted to visualize the relationship. The SAC and WRA data were analyzed separately, which included all datasets for UZC (4) and SZC (3). As such, 12 models for WRA and SAC were computed and assessed.

### *Post-hoc* analyses

*Post-hoc* analyses were undertaken to understand the relationship between UZC and SZC and to further explore potential cut-off points for zinc status from the UZC data. First, a regression model was used to explore the impact of inflammation on UZC. The regression model used log-transformed UZC data as the independent variable and the inflammatory markers c-reactive protein (CRP) and α-1 acid glycoprotein (AGP) as predictor variables. This was done for both SAC and WRA and all the three UZC data types.

Second, attempts were made to achieve objective 2 by using values based on estimated average urinary zinc losses. These values were estimated from published literature by expert panels for the estimation of zinc physiological requirements (PR) during the process of arriving at dietary zinc recommendations using the factorial approach. The hypothesis is that if dietary zinc intake is not sufficient to meet the PR, then homeostatic adjustments to preserve zinc occur, including reduced zinc losses in urine (2). Urinary zinc losses below these thresholds may thus be indicative of inadequate zinc intake. Age and gender-specific values for zinc loss *via* urine were used for SAC (0.1 mg/day for SAC aged 5–6, 0.2 mg/day for SAC aged 7–10 years, 0.3 mg/day for male and female SAC aged 11–14 years, after rounding), and for women, the threshold of 0.3 mg/day WRA (25) was used. The values were published by European Food Safety Authority (EFSA), but are based on reference bodyweight values from a study by van Buuren et al. (for SAC) (26), and multiple individual studies for WRA (27–32). For control, the threshold value for zinc loss that is recommended by the Food and Nutrition Board (FNB)/ Institute of Medicine (IOM) of 0.44 mg/day in WRA, was also tested. This analysis was done using the hydration-adjusted data only, as it implied conversion of the spot urine samples to equivalent 24-h values. The estimated prevalence of observations with reduced dietary intake was then compared with the estimated prevalence of serum zinc deficiency using a Chi-squared test of proportions.

### Statistical inferences

For all analyses, a *p*-value of 0.05 was considered statistically significant and prevalence estimates are reported with a 95% confidence interval. All point estimates were weighted using the household-level sampling weights which were derived from the survey metadata.

### Data access and ethical approval

Urinary zinc data was accessed from Phiri et al. (21) whereas the serum zinc data was accessed from the DHS program database following approval of the request for use (May 23rd, 2019). Ethical approval for this secondary data analysis was obtained from the College of Medicine Research Ethics Committee (COMREC), protocol number P.01/20/2915 (March 11th, 2020). COMREC is affiliated with Kamuzu University of Health Sciences, previously known as the College of Medicine in Malawi.

## Results

### Participants and sample characteristics

Spot urine zinc analysis was conducted for *n* = 1,406 participants (WRA: *n* = 741 and SAC: *n* = 665), whereas serum zinc analysis was conducted for *n* = 1,488 (WRA: *n* = 745 and SAC: *n* = 743). After both datasets were cleaned and matched *n* = 1,301 observations remained of which 660 were WRA and 641 were SAC as shown in Table 1 and Supplementary Figure S1.

**Table 1:** Participant demographics and geometric mean +SD values for urine and plasma biochemical parameters measured in women and school-age children in the 2015–2016 Malawi national micronutrient survey.

	**SAC**	**WRA**
*N*	641	660
Male	309	-
Female	332	-
SZC_unadj_ (Mean ± SD) μg/dL	60.69 ± 1.29	58.96 ± 1.25
CRP (Mean ± SD) mg/L	0.88 ± 6.47	0.73 ±4.59
AGP (Mean ± SD) g/L	0.83 ± 1.65	0.62 ± 1.53
UZC_unadj_ (Mean ± SD) μg/L	276.99 ± 2.64	278.93 ± 2.92
Cr (Mean ± SD) g/L	0.64 ± 2.33	1.00 ± 2.06
Osm (Mean ± SD) mOsm/L	472.14 ± 1.76	534.18 ± 1.67
Sg (Mean ± SD)	1.02 ± 1.01	1.01 ±1.00

*WRA, Women of reproductive age; SAC, school-age children; SZC_unadj_, unadjusted serum zinc concentration; CRP, c-reactive protein; AGP, Alpha-1 acid glycoprotein; UZC_unadj_, Unadjusted urine zinc concentration; Cr, Urine Creatinine concentration; Osm, urine Osmolarity concentration; Sg, Urine specific gravity, all mean and SD values are geometric means*.

For SZC, an upper threshold of 125 μg/dl was used (33), beyond which samples were suspected to have been contaminated and deemed as outliers. All urine samples were deemed of good quality and not too dilute for the element analyses because none of the observations had both creatinine and Sg levels that were below the thresholds of dilution. Only two observations (one in each demographic group) had creatinine values below 0.05 g/L and there were no observations with Sg below 1.001.

### Adjustment for urine data for hydration

**Figure 1:**
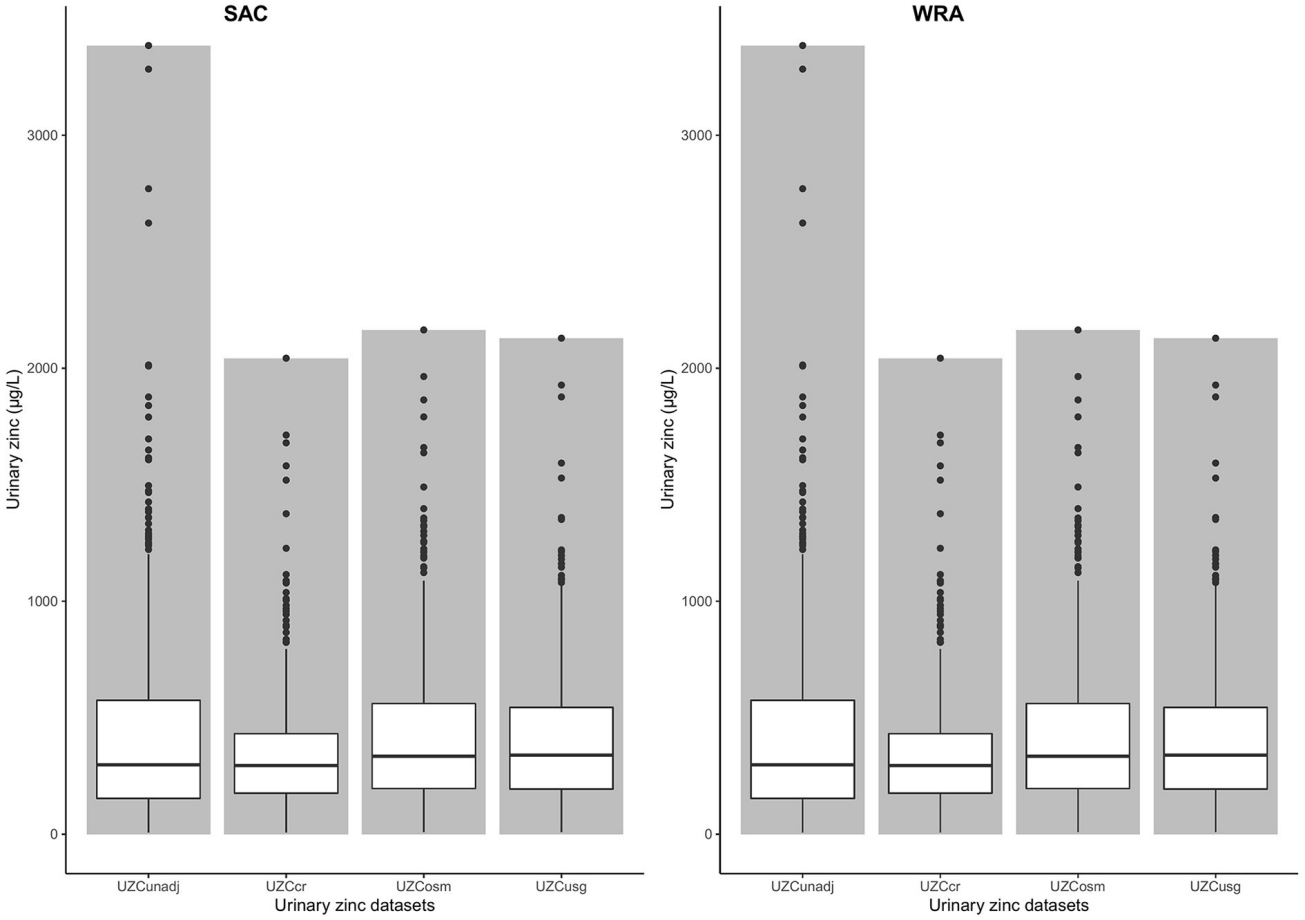
Bar chart with box-plots for urine zinc concentration values and means among women of reproductive age and school-age children, before and after adjustment for hydration status in the 2015–2016 Malawi national micronutrient survey. UZC_unadj_: unadjusted urine zinc concentration, UZC_cr_: Creatinine adjusted urine zinc concentration, UZC_osm_: Osmolarity adjusted urine zinc concentration, UZC_sg_: Specific gravity adjusted urine concentration, SAC: School-age children, WRA: Women of reproductive age.

The mean Cr, Osm, and Sg values that were used for the hydration adjustments are presented in Table 1. There were higher mean concentrations of Cr and Osm in WRA compared to SAC. The data for both SAC and WRA was not normally distributed before or after hydration adjustment, based on Shapiro–Wilk tests which gave extremely low *p*-values (*p* < 0.01). The mean UZC in SAC decreased following adjustment, from 410.95 to 396.39 and 385.51 μg/L for Osm and Sg, respectively. These changes were not significant, based on Wilcoxon signed-rank tests. Adjusting for creatinine, significantly increased the mean UZC of SAC to 500.23 μg/L (*p* < 0.01; Figure 1). Hydration adjustment of the UZC of WRA led to decreases in the UZC for all three methods. Wilcoxon signed-rank tests indicated that the hydration adjusted values for WRA were significantly lower than the unadjusted values (UZC_cr_; *p* < 0.01, UZC_osm_; *p* < 0.01, UZC_sg_; *p* = 0.01).

### Linear mixed-effects model results of SZC and UZC data

As shown in Table 2, in the null model of SZC, variance components were very small for SAC for hydration-adjusted and unadjusted data. This was true for all three sampling levels and the largest (albeit still small) variance was observed between individuals within the household level for the unadjusted SZC data. The addition of urinary zinc as random terms to the SZC model did not improve the efficiency of the model for SAC. This is based on the inability of the UZC data to reduce the variance component of the null model. This observation was true for all model combinations of all three SZC types and all four UZC types. A reduction in the variance of the adjustment for inflammation reduced the variance slightly but the change was negligible. No variation in the SZC data could thus be explained by the addition of UZC data of any hydration adjustment type.

**Table 2:** Variance components for the linear mixed-effects model (random effects and fixed effects) with serum zinc concentration as the dependent variable and urine zinc concentration as the predictor variable for school-age children in the 2015–2016 Malawi national micronutrient survey.

**Model predictors**		**Between cluster level**		**Between household level**		**Between individuals within household level**		**Fixed effects point estimates**			
		**Variance**	**Adjusted** *R*^2^	**Variance**	**Adjusted** *R*^2^	**Variance**	**Adjusted** *R*^2^	**Coefficient**	**Std. error**	***t*** **-value**	***p*** **-value**
SZC_unadj_	Null	0.01	-	0.01	-	0.04	-	4.09	0.02	257.66	<0.01
	UZC_unadj_	0.01	0.12	0.01	0.14	0.04	0.05	0.06	0.01	6.76	<0.01
	UZC_cr_	0.01	0.16	0.01	−0.10	0.04	0.07	0.09	0.01	6.76	<0.01
	UZC_osm_	0.01	0.18	0.01	0.05	0.04	0.07	0.09	0.01	7.25	<0.01
	UZC_sg_	0.01	0.21	0.01	0.03	0.04	0.07	0.09	0.01	7.90	<0.01
SZC_icf_	Null	0.01	-	0.01	-	0.04	-	4.12	0.01	264.95	<0.01
	UZC_unadj_	0.01	0.12	0.01	0.17	0.04	0.05	0.06	0.01	7.05	<0.01
	UZC_cr_	0.01	0.16	0.01	−0.01	0.04	0.07	0.09	0.01	6.99	<0.01
	UZC_osm_	0.01	0.18	0.01	0.07	0.04	0.07	0.09	0.01	7.69	<0.01
	UZC_sg_	0.01	0.20	0.01	0.14	0.04	0.07	0.10	0.01	8.13	<0.01
SZC_br_	Null	0.01	-	0.01	-	0.04	-	4.16	0.01	269.12	<0.01
	UZC_unadj_	0.01	0.11	0.01	0.16	0.04	0.05	0.06	0.01	6.83	<0.01
	UZC_cr_	0.01	0.15	0.01	−0.00	0.04	0.07	0.09	0.01	6.94	<0.01
	UZC_osm_	0.01	0.18	0.01	0.07	0.04	0.07	0.09	0.01	7.49	<0.01
	UZCs_g_	0.01	0.20	0.01	0.14	0.04	0.07	0.10	0.01	7.93	<0.01

*SZC_unadj_, Unadjusted serum zinc concentration; SZC_icf_, Internal correction factor adjusted serum zinc concentration; SZC_br_, serum zinc concentration that is adjusted by Biomarkers Reflecting Inflammation and Nutritional Determinants of Anemia (BRINDA) unadjusted urine zinc concentration; UZC_unadj_, Unadjusted urine zinc concentration; UZC_cr_, Creatinine adjusted urine zinc concentration; UZC_osm_, Osmolarity adjusted urine zinc concentration; UZC_sg_, Specific gravity adjusted urine concentration*.

**Figure 2:**
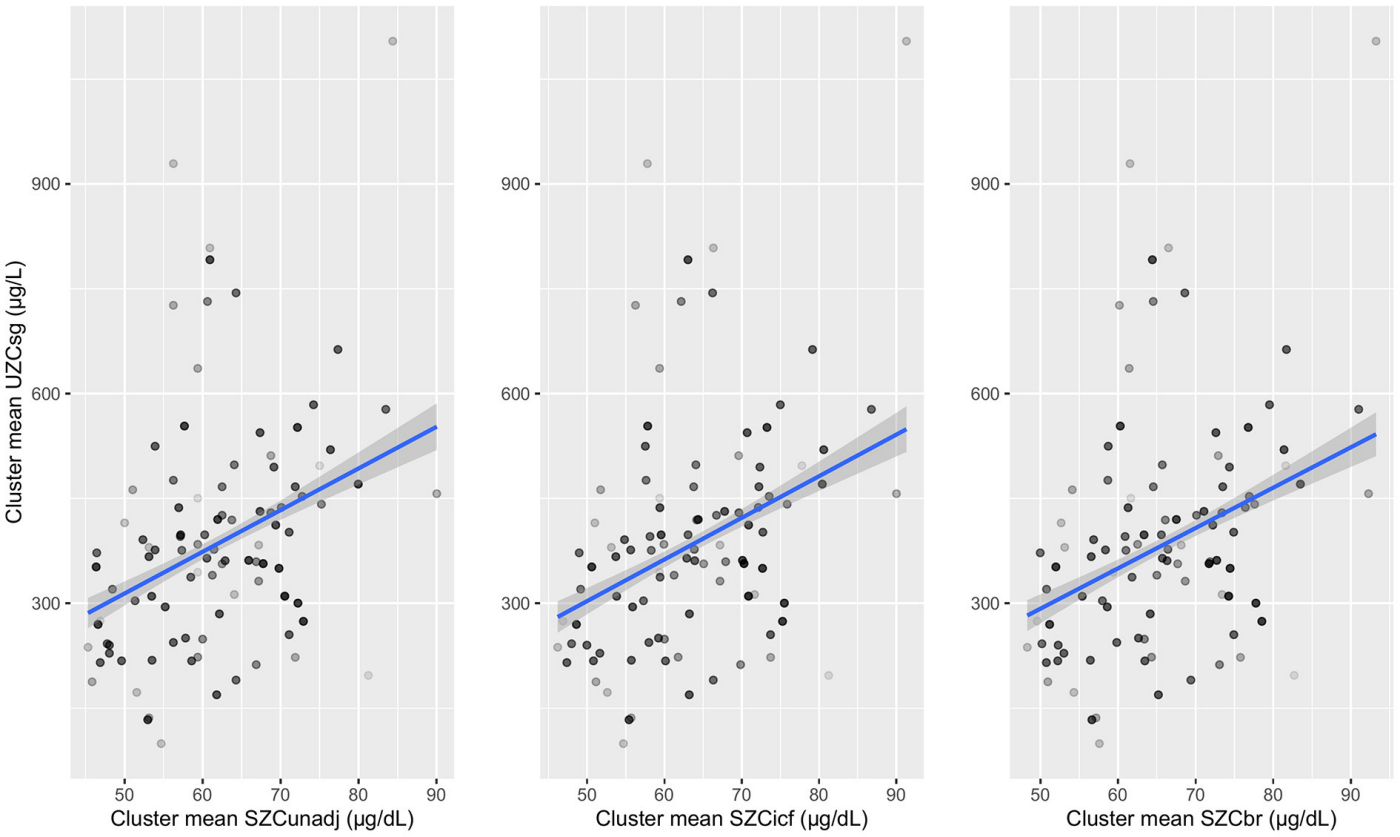
Cluster means of urinary zinc concentrations adjusted for specific gravity (UZC_sg_) plotted against serum zinc concentrations which are unadjusted and adjusted for inflammation (using internal correction factor and biomarkers reflecting inflammation and nutritional determinant of anaemia methods) in school-aged children. UZC_sg_: Urinary zinc concentration adjusted for specific gravity, SZC: serum zinc concentration, SZC_unadj_: Unadjusted serum zinc data, SZC_icf_: Serum zinc concentration adjusted using internal correction factor method (ICF), SZC_br_: Serum zinc concentration adjusted using biomarkers reflecting inflammation and nutritional determinant of anaemia method (BRINDA).

For SAC, the adjusted *R*^2^ was largest at between cluster level compared to between household or between individual within household levels, for all three SZC types. The adjusted *R*^2^ increased when UZC was adjusted for hydration and the largest value of adjusted *R*^2^ (0.21) is observed for UZC_sg_ as a predictor for unadjusted SZC data. This largest adjusted *R*^2^ was observed at the cluster level and the cluster means for this data type were plotted against those of the SZC data types (Figure 2). A linear relationship can be seen between the cluster means of UZC_sg_ and the SZC data types in all three graphs in Figure 2. Overall, adjustment for inflammation did not improve the relationship between SZC and UZC in SAC data.

For WRA, the variance between household levels was extremely low for all the models; as such, the adjusted *R*^2^ at this level could not be interpreted (Table 3). At between cluster level, the addition of UZC_cr_ to the null model did not affect the variance and the adjusted *R*^2^ for this data type was equal to 0. The other data types also had very low adjusted *R*^2^-values which indicated a lack of improved efficiency despite the addition of the UZC data to the null model. Similar results were observed among individuals, within the household level where low adjusted *R*^2^-values were observed. Adjustment for hydration did not have any effect on the variation of the null model, for all SZC data types at the varying levels.

**Table 3:** Variance components for the linear mixed-effects model (random effects and fixed effects) with serum zinc concentration and the dependent variable and urine zinc concentration as the predictor for women in the 2015–2016 Malawi national micronutrient survey.

**Model predictors**		**Between cluster level**		**Between household level**		**Between individuals within household level**		**Fixed effects point estimates**			
		**Variance**	**Adjusted** *R*^2^	**Variance**	**Adjusted** *R*^2^	**Variance**	**Adjusted** *R*^2^	**Coefficient**	**Std. error**	***t*** **-value**	***p*** **-value**
SZC_unadj_	Null	0.01	-	0.00	-	0.04	-	4.07	0.01	269.86	<0.01
	UZC_unadj_	0.02	0.04	0.00	0.00	0.04	0.04	0.05	0.01	5.23	<0.01
	UZC_cr_	0.02	−0.00	0.00	0.00	0.04	0.04	0.06	0.01	4.82	<0.01
	UZC_osm_	0.02	0.04	0.00	0.00	0.04	0.05	0.06	0.01	5.82	<0.01
	UZC_sg_	0.02	0.05	0.00	0.00	0.04	0.06	0.07	0.01	6.28	<0.01
SZC_icf_	Null	0.02	-	0.00	-	0.04	-	4.07	0.01	271.42	<0.01
	UZC_unadj_	0.02	0.04	0.00	0.00	0.04	0.04	0.04	0.01	5.07	<0.01
	UZC_cr_	0.02	−0.00	0.00	0.00	0.04	0.04	0.06	0.01	4.80	<0.01
	UZC_osm_	0.02	0.05	0.00	0.00	0.04	0.05	0.06	0.01	5.71	<0.01
	UZC_sg_	0.02	0.05	0.00	0.00	0.04	0.05	0.07	0.01	6.18	<0.01
SZC_br_	Null	0.02	-	0.00	-	0.06	-	4.09	0.01	275.31	<0.01
	UZC_unadj_	0.02	0.04	0.00	0.98	0.06	0.02	0.05	0.01	5.31	<0.01
	UZC_cr_	0.02	−0.00	0.00	0.61	0.06	0.02	0.06	0.01	4.84	<0.01
	UZC_osm_	0.02	0.05	0.00	0.83	0.06	0.03	0.06	0.01	5.93	<0.01
	UZCs_g_	0.02	0.05	0.00	0.98	0.06	0.03	0.07	0.01	6.31	<0.01

*SZC_unadj_, Unadjusted serum zinc concentration; SZC_icf_, internal correction factor adjusted serum zinc concentration; SZC_br_, serum zinc concentration that is adjusted by Biomarkers Reflecting Inflammation and Nutritional Determinants of Anemia (BRINDA) unadjusted urine zinc concentration; UZC_unadj_, Unadjusted urine zinc concentration; UZC_cr_, Creatinine adjusted urine zinc concentration; UZC_osm_, Osmolarity adjusted urine zinc concentration; UZC_sg_, Specific gravity adjusted urine concentration*.

### *Post-hoc* analyses

The multivariate linear regression revealed no significant relationship between inflammatory markers and urinary zinc data across all the models and for SAC (Table 4). For WRA, there was a significant relationship between AGP and UZC_unadj_, but with a coefficient of 0. Thus, no variation in UZC data of all types could be explained by the inflammatory markers.

**Table 4:** Fixed effects inferential of a linear mixed model (random effects and fixed effects) with urine zinc concentration as the dependent variable and inflammatory markers as the predictor variables for school-age children and women in the 2015–2016 Malawi national micronutrient survey.

**SAC**								
	**CRP**				**AGP**			
**Data type**	**Coefficient**	**Std. error**	***t*-value**	***p*-value**	**Coefficient**	**Std. error**	***t*-value**	***p*-value**
UZC_unadj_	−0.01	0.08	−0.11	0.92	−0.02	0.02	−0.97	0.33
UZC_cr_	−0.05	0.13	−0.43	0.67	0.03	0.03	0.97	0.33
UZC_osm_	0.02	0.11	0.14	0.89	0.01	0.03	0.27	0.78
UZCs_g_	−0.11	0.12	−0.91	0.37	−0.01	0.03	−0.36	0.72
**WRA**								
	**CRP**				**AGP**			
UZC_unadj_	0.01	0.06	0.15	0.88	0.00	0.02	0.10	0.00
UZC_cr_	0.01	0.09	0.09	0.93	−0.01	0.02	−0.54	0.58
UZC_osm_	0.06	0.08	0.74	0.46	0.00	0.02	0.18	0.85
UZCs_g_	0.00	0.08	0.02	0.99	−0.01	0.02	−0.43	0.67

*UZC_unadj_, Unadjusted urine zinc concentration; UZC_cr_, Creatinine adjusted urine zinc concentration; UZC_osm_, Osmolarity adjusted urine zinc concentration; UZC_sg_, Specific gravity adjusted urine concentration; SZC_unadj_, Unadjusted serum zinc concentration; SZC_icf_, internal correction factor adjusted serum zinc concentration; SZC_br_: UZC_unadj_, serum zinc concentration that is adjusted by Biomarkers Reflecting Inflammation and Nutritional Determinants of Anemia (BRINDA) unadjusted urine zinc concentration; SAC, school-age children; WRA, Women of reproductive age; CRP, c-reactive protein; AGP, Alpha-1 acid glycoprotein*.

For SAC, the EFSA threshold of urinary zinc excretion (0.1–0.3 mg/day) gave low estimates of zinc deficiency compared to SZC for all the data types. The lowest estimate was observed with UZC_cr_ data and the highest, with UZC_unadj_ (Table 5). There was no overlap in the confidence intervals between the UZC and the SZC estimates.

**Table 5:** Prevalence estimates of zinc deficiency in urinary zinc concentration, and serum zinc concentration for school-age children and women in the 2015–2016 Malawi national micronutrient survey.

	**SAC**	**WRA**	
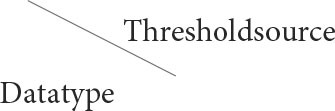	**EFSA**	**EFSA**	**FNB/IOM**
		**Prevalence estimate % (95% CI)**		
UZC_unadj_	33 (27; 40)	50 (44; 56)	65 (59; 70)
UZC_cr_	17 (13; 22)	49 (42; 56)	75 (68; 80)
UZC_osm_	26 (21; 32)	47 (41; 53)	64 (58; 69)
UZC_sg_	25 (19; 32)	45 (39; 51)	63 (58; 69)
SZC	55 (48; 61)	62 (55; 69)	
SZC_icf_	50 (43; 56)	61 (54; 67)	
SZC_br_	43 (36; 50)	59 (52; 66)	

*SAC, school-age children; WRA, women of reproductive age; EFSA, European food safety authority; FNB/IOM, Food and nutrition board/Institute of Medicine; UZC_unadj_, Unadjusted urine zinc concentration; UZC_cr_, Creatinine adjusted urine zinc concentration; UZC_osm_, Osmolarity adjusted urine zinc concentration; UZC_sg_, Specific gravity adjusted urine concentration; SZC_unadj_, Unadjusted serum zinc concentration; SZC_icf_, internal correction factor adjusted serum zinc concentration; SZC_br_, serum zinc concentration that is adjusted by Biomarkers Reflecting Inflammation and Nutritional Determinants of Anemia (BRINDA)*.

For WRA, the urinary zinc threshold value of 0.3 mg/day, suggested by EFSA gave prevalence estimates of zinc deficiency ranging from 45 to 50% (Table 5). These were lower than the estimates of zinc deficiency as identified by SZC before and after adjusting for inflammation. The lower tail of the confidence intervals for all the EFSA threshold estimates for SZC had an overlap with the upper tails of the confidence intervals of UZC_unadj_, UZC_cr_, and UZC_osm_. The prevalence estimates from the threshold by FNB/IOM (0.44) were higher than the estimates for SZC. These estimates had a slight overlap with the higher tail of the confidence intervals of the SZC estimates.

## Discussion

The assessment of zinc in humans at the population level remains a challenge due to the lack of suitable zinc biomarkers (2). Spot urine is a good candidate for determining zinc deficiency at the population level, but the lack of threshold for defining deficiency in this biomarker poses a limitation to its adoption and use. This current study aimed to use paired UZC and SZC data, to identify thresholds in urine that are equivalent to those that are used to define zinc deficiency in serum. This was dependent on a strong relationship between these two biomarkers to allow for the adoption of the thresholds.

The paired UZC and SZC data showed a poor correlation in a linear mixed-effects model of SAC and WRA data from a population-based survey. The correlation analysis factored in the sampling design to take into account the differences between clusters, differences between households, and differences between individuals within a household. Before the analysis, the UZC data was adjusted for hydration using three different methodologies, and the SZC was adjusted for effects of inflammation using two different methods. All these confounder corrections were applied to correct for biological differences that occur due to infection and variation in hydration patterns and to optimize the performance of the UZC data in the correlation test. Various factors may have contributed to the poor correlation despite the adjustments for hydration and inflammation. A previous study that used this same SZC data identified possible data quality issues across all demographic groups which resulted in clumping of the SZC values, i.e., more than 50 observations with the same SZC value (9), possibly due to factors associated with limits of detection.

As shown in Table 3, about 20% of the variation in the SZC data for SAC could be explained by the UZC_sg_ data and this was true for unadjusted as well as inflammation-adjusted SZC data. The correlation values were similar but lower for the other hydration adjustment data. Unadjusted UZC data for SAC also showed a stronger relationship at between household level, but this was not the case between individuals. For WRA, the correlation was weak for all the UZC data types against all the SZC data types. This implies that UZC may be a better population-level marker for SAC than it is for WRA. It is not clear from the results, why the correlation strength differed between SAC and WRA, but physiological differences between these two groups could have contributed to this. Similar results were found by Phiri et al. (21), who assessed spatial variation in this same spot urine zinc data. Their results showed spatial dependence between clusters in UZC for SAC but not for WRA indicating the possible influence on the SAC-UZC by environmental factors such as soil type which has been reported to play an important role in the zinc content of locally grown crops (34).

Various adjustment methods were applied to try and optimize the performance of the linear regression model. Acute infection and inflammation are common factors that affect the homeostasis of various elements in the body as such, in participants with high levels, of inflammation, it is necessary to adjust for their effects. The serum zinc data were adjusted to account for inflammation but this had little effect on the regression statistics. Unadjusted SZC data for SAC had similar and weak correlations at all cluster levels, for all UZC types. The urinary data was also corrected for hydration but results showed that unadjusted data had a stronger correlation with SZC than creatinine or osmolality-adjusted urine data, for the SAC analysis (Table 3).

Hydration adjustment improved the relationship between UZC and SZC for SAC, the strongest being UZC_sg_, followed by UZC_osm_, and finally, UZC_cr_, which indicates clear effects of hydration on the data. There was no discernible pattern of the hydration adjustment methods for WRA and there seemed to be many variations, especially for creatinine-adjusted data. For this demographic group, the performance of the unadjusted UZC data did not differ from UZC_sg_ and UZC_osm_. This indicates possible challenges with hydration adjustments in specific demographic groups, which may have affected the correlation. Although analyses of 24-h urine samples do not need to be adjusted for hydration status or creatinine concentration, they also have poor correlations with SZC data indicating that urine itself may just be a poor biomarker for zinc deficiency regardless of hydration effects (35). However, considering that these analyses were done in the 1970s, new research with improved assay technologies may be warranted.

The lack of agreement between the existing adjustment methods for both inflammation and hydration corrections implies the need to improve the performance of the individual methods. This is a limitation of their use when adjusting various data and could have contributed to the poor linear regression performance. Measurement error can also contribute to the poor performance of a linear regression equation. In this analysis measurement error in either of the two main datasets (UZC and SZC), the inflammatory data (CRP and AGP) or the hydration indicators (Creatinine, Osmolality, Urine specific gravity) could have caused this. However, the large sample sizes control for random error, and available QC data for most of the datasets did not indicate any systematic errors (36).

The weak correlation between UZC and SZC indicates that UZC may have limited value as a biomarker of population-level zinc deficiency even for SAC. There is thus still a need to find a biomarker for zinc that is sensitive, specific, and easily accessible. SZC is the most widely used biomarker for zinc, however, it requires venipuncture blood which can only be collected by skilled professionals, and requires specific techniques. Furthermore, extraction of the serum should be done in a sterile environment which is difficult to adhere to in large scale surveys where samples are collected in the field (37). A biomarker with easier sample collection and management (such as UZC) is therefore preferred. It would allow the collection of samples from large sample sizes with fewer technical limitations. A *post-hoc* analysis was employed to further explore the relationship between UZC and SZC data. Acute inflammation has been previously shown to cause hyperzincuria (38), which can result in a poor correlation between UZC and SZC since SZC is a negative acute-phase reactant (39, 40). There were high levels of inflammation in SAC (34%), and some inflammation in WRA at 14% (18), however no statistically significant associations with UZC in either the WRA or SAC groups were found.

An alternative approach to identify thresholds for zinc deficiency in urine was explored using average urinary zinc losses in healthy individuals (14, 15) as putative cut-off values. The EFSA thresholds yielded lower prevalence values for zinc deficiency than those calculated using SZC in both SAC and WRA groups. These zinc loss values (0.3 mg/day for WRA and 01.0–0.3 mg/day for SAC) by EFSA (15) were determined by using the factorial approach, based on endogenous zinc losses as assessed by multiple individual studies, most of which were done in the early 2000s.

Other panels have also estimated mean urinary zinc loss and found similar values. For instance, the World Health Organization (WHO) estimates 0.3 mg/day (41) whereas, FNB/IOM estimated urinary zinc losses to be 0.44 mg/day in women, based on studies mostly conducted in the early 1990s (42). This value was also adopted by the IZiNCG (14). When applied to the WRA data the value suggested by FNB/IOM and IZiNCG gave prevalence estimates that were slightly higher than those obtained by SZC. For UZC_Osm_ and UZC_Usg_, the confidence intervals of the prevalence estimates were in the same range as the SZC estimates. With novel primary research, there may be a possibility of determining appropriate thresholds indicating zinc deficiency based on zinc loss, or other means.

The main limitation of this study is that it failed to identify the optimal cut-off point in UZC for estimating the risk of zinc deficiency. This was because there was a poor correlation between the UZC and SZC datasets, which could have occurred because of the quality of the lab results. Examination of the quality control data indicated that there was no measurement error in all the lab analyses of the main datasets. However, for SZC, the assay method used (atomic emission spectrometry) has a higher limit of detection, compared to modern methods such as ICP-MS which was used for UZC analysis. In populations with low levels of zinc deficiency, as the sample for this study, higher limits of detection could lead to data clumping on the lower end of the dataset (9), thus leading to differences in paired data. In the future, high-precision assay instruments should be used for element analysis to ensure that paired data results are measured similarly.

Paired spot urinary and plasma/serum mineral concentrations have been used to determine probable thresholds for mineral deficiencies including vitamin A and selenium. However, for zinc, the poor correlation between these two datasets in a national sample of the Malawi population, despite correcting for multiple confounders, raises questions about whether this approach is appropriate for determining the threshold indicating deficiency for this mineral. The slightly better correlation between UZC and SZC for SAC only, at the cluster level, indicates that spot urine samples may have value at the population level for SAC but not WRA. Nonetheless, it is not clear to what extent this potential can be useful for determining zinc status. An alternative approach to estimate zinc deficiency prevalence—using mean urinary zinc excretion as a cut-off point for deficiency—also gave conflicting results compared to estimates based on SZC, which highlights a need for primary research on appropriate thresholds of zinc concentration in spot urine. Further investigation is also required to understand the value of spot urine zinc at the population level. It is also of great interest to explore if the spatial patterns that are observed in SAC but not WRA, can also be observed in other demographic groups. This could further inform on the applications of spot urine zinc in population zinc status measurement.

## Data availability statement

Publicly available datasets were analyzed in this study. This data can be found here: Serum zinc data can be obtained from https://dhsprogram.com/data/dataset/Malawi_Standard-DHS_2015.cfm?flag=0 and Urinary zinc data, can be obtained from the authors of https://link.springer.com/article/10.1007/s10653-020-00700-5.

## Ethics statement

The studies involving human participants were reviewed and approved by Malawi College of Medicine Research Ethics Committee (COMREC), Protocol Number P.01/20/2915. Written informed consent to participate in this study was provided by the participants' legal guardian/next of kin.

## Author contributions

Conceptualization and writing—review and editing: BL, RL, NL, and EJ. Data curation: BL and RL. Formal analysis, investigation, and writing—original draft: BL. Funding acquisition and project administration: EJ. Methodology: BL, RL, and NL. Supervision: KM and JP. Validation: RL. All authors contributed to the article and approved the submitted version.

## Funding

This work was supported, in whole or in part, by the Bill & Melinda Gates Foundation (INV-009129). Under the grant conditions of the Foundation, a Creative Commons Attribution 4.0 Generic License has already been assigned to the Author Accepted article version that might arise from this submission. The funder had no role in the design, execution, analysis, or interpretation of the data.

## Conflict of interest

The authors declare that the research was conducted in the absence of any commercial or financial relationships that could be construed as a potential conflict of interest.

## Publisher's note

All claims expressed in this article are solely those of the authors and do not necessarily represent those of their affiliated organizations, or those of the publisher, the editors and the reviewers. Any product that may be evaluated in this article, or claim that may be made by its manufacturer, is not guaranteed or endorsed by the publisher.
