# Supplementary material for: The potential of spot urine as a biomarker for zinc assessment in Malawian children and adults
Source: Front Nutr. 2022 Jul 22;9:890209. doi: 10.3389/fnut.2022.890209 (PMC9355503; doi:10.3389/fnut.2022.890209)
Supplement: Supplementary file 1 [file Data_Sheet_1.docx]

**Figure 1 Supplementary figure showing the study participant selection flow chart of data on women of reproductive age (WRA) and school-age children (SAC) in the 2012-2016 Malawi national micronutrient survey**
